# Evaluation of a Primary Care Weight Management Program in Children Aged 2–5 years: Changes in Feeding Practices, Health Behaviors, and Body Mass Index

**DOI:** 10.3390/nu11030498

**Published:** 2019-02-27

**Authors:** Jared M. Tucker, Renee DeFrang, Julie Orth, Susan Wakefield, Kathleen Howard

**Affiliations:** 1 Healthy Weight Center, Helen DeVos Children’s Hospital, 35 Michigan, Suite 1800 MC232, Grand Rapids, MI 49503, USA; 2 Department of Pediatrics and Human Development, Michigan State University, Life Sciences Bldg.1355 Bogue St., B240, East Lansing, MI 48824, USA; kathleenhoward@comcast.net; 3 We Are For Children, LLC, 877 Forest Hill Ave SE, Grand Rapids, MI 49546, USA; reneeadefrang@gmail.com (R.D.); julie.orth1@gmail.com (J.O.); swakefield@weareforchildren.com (S.W.); 4 Forest Hills Pediatrics, 877 Forest Hill Ave SE, Grand Rapids, MI 49546, USA

**Keywords:** pediatric obesity, preschool children, non-responsive feeding, meal environment, parenting

## Abstract

Background: Primary care offers a promising setting for promoting parenting practices that shape healthy eating and physical activity behaviors of young children. This study assessed the impact of a parent-based, primary care intervention on the feeding habits, health behaviors, and body mass index (BMI) of 2–5 year olds with elevated or rapidly-increasing BMI. Methods: Four private pediatric offices in West Michigan were assigned as control (*n* = 2) or intervention (*n* = 2) sites based on patient load and demographics. Treatment families were recruited at well-child visits to receive physician health-behavior counseling and four visits with a registered dietitian nutritionist (RDN) over a 6-month period. Intervention outcomes were age- and sex-specific BMI metrics, including BMI z-scores and percent of the 95th percentile (%BMI_p95_), the Family Nutrition and Physical Activity survey (FNPA), and the Feeding Practices and Structure Questionnaire (FPSQ). Results: Of 165 enrolled families, 127 completed follow-up measures (77% retention). Mean (±SD) FNPA scores improved in treatment vs. control (4.6 ± 4.6 vs. 0.1 ± 4.2; *p* < 0.001), and screen time (h/day) decreased (−0.9 ± 1.8 vs. 0.3 ± 1.1; *p* < 0.001). Non-responsive feeding practices (i.e., reward for behavior (*p* = 0.006) and distrust in appetite (*p* < 0.015)) and structure-related feeding practices (structured meal timing (*p* < 0.001)) improved in treatment parents vs. controls. Reductions in child BMI measures did not differ between groups. Conclusions: Families with preschool children participating in a low-intensity, primary care intervention improved obesogenic health behaviors, parent feeding habits, and child screen time, but not child adiposity. Future research should assess the sustainability of these family lifestyle improvements, and evaluate their future impact on the health and development of the children.

## 1. Introduction

The epidemic of childhood obesity, along with its immediate and long-term health consequences, makes this medical condition one of the most important health issues facing the U.S. and much of the world [1,2,3,4,5]. Previous research indicates that the risk of obesity during adolescence and adulthood is largely influenced during early childhood [6]. In the United States, almost one in three kindergarteners with overweight will have obesity by the time they are in eighth grade vs. 7.9% of kindergarteners with normal weight [7]. This represents a four-fold difference in the risk of developing obesity based on weight status in early childhood. In addition, several growth-related factors in youth between 2–5 years old have been identified as risk factors for later obesity, including early elevated body mass index (BMI) and rapid BMI gains (i.e., “catch-up” growth) and an early adiposity rebound [8,9,10,11,12]. Thus, early childhood may represent a window of opportunity for establishing healthy weight trajectories that can be maintained into adolescence and adulthood.

Research suggests that parent feeding practices may provide a promising target for interventions seeking to influence the weight and weight-related eating habits of young children. In particular, non-responsive feeding behaviors (i.e., those that use food as a mechanism to control children’s eating and behavior rather than relying on hunger) have been linked to increases in overeating, emotional eating and adiposity [13,14,15]. Furthermore, initial evidence indicates that non-responsive feeding behaviors can be positively influenced through educational interventions [16,17,18,19].

Primary care may provide a favorable setting for obesity health behavior interventions that target parent feeding practices and other health behaviors in families with young children due to the provider’s rapport and credibility with the family, and a mechanism for consistent contact through regular well-child visits. Despite these advantages, pediatric clinicians often lack the training and time needed to implement intensive behavioral treatment for their patients with obesity. Thus, interventions are needed within the primary care setting that can provide clinician training and include clinicians in the treatment and follow up of patients, while also removing the bulk of the intervention workload from primary care providers. 

To date, few pediatric weight management programs in primary care settings have been evaluated, and even fewer have been studied that target both parent feeding behaviors as well as child physical activity and sedentary behaviors. The ‘We Are For Children’ healthy lifestyles intervention is a 6-month behavior-based obesity treatment program administered by both pediatric clinicians and dietitians in the primary care setting. This investigation evaluated the ‘We Are For Children’ program using a quasi-experimental design to compare changes in parental feeding habits, child health behaviors, and child BMI among families of 2–5 year-old children at risk of overweight or obesity. 

## 2. Methods

### 2.1. Study Design and Setting

We Are for Children, LLC (WAFC), is a collaboration of non-profit pediatric practices in West Michigan formed to help members respond to healthcare needs. Four primary care pediatric offices from WAFC were selected to participate in the current study based on site interest and capacity. Participating pediatric offices were then matched into pairs based on similarities in office size (i.e., physician FTEs and patient load) and patient demographics characteristics, after which one of each matched pair was assigned as a treatment or control site. 

### 2.2. Participant Recruitment, Enrollment, and Protection

Participants were comprised of families with children aged 2–5 years who attended one of the four participating primary care pediatric offices. Patient inclusion criteria were screened by office staff during well-child office visits. Patient age, height and weight were assessed and entered into electronic medical records in order to plot current age- and sex-specific body mass index (BMI). Those within the appropriate age range were screened for BMI ≥85th percentile (overweight), or a rapid BMI increase such that ≥2 BMI percentile lines were crossed during the past year on a standard CDC growth chart, excluding the 5th percentile (e.g., 10th and 25th, 25th and 50th, or 50th and 75th). This rate of increase is equivalent to ≥0.67 standard deviations per year (BMI z-score), which has been shown, when occurring in this age group, to increase risk of adult obesity [20]. Eligible patients/parents were informed of the study and given a flyer explaining basic information about the research project during their well-child visit. Families interested in participating were given the informed consent document and study surveys to complete during the visit. Participants from intervention sites were contacted by a registered dietitian nutritionist (RDN) to answer any questions about the intervention and to schedule the first RDN visit. All participant assessments, RDN treatment visits, and pediatrician-family interactions took place in the primary care pediatric office. There was no cost to participate in the study, and both treatment and control families were given a $20 gift card for completing the study. All study procedures were approved by the Spectrum Health Institutional Review Board, and written consent was obtained from all parents participating in the research. 

### 2.3. Treatment Groups

Control—Control parents signed the informed consent document and then completed all baseline assessments during their child’s medical visit. Control participants then received their usual medical care. Follow-up evaluations, including child anthropometry and completion of study surveys were assessed approximately six months later during a second office visit. For children <3 years of age at baseline, follow up visits coincided with their subsequent well-child visit (i.e., 30–month or three-year appointment) as per AAP visit frequency recommendations [21]. For patients ≥3 years of age, families attended a separate office visit six months after their baseline visit in order to complete follow-up study assessments. 

Intervention—The study intervention included two primary components: (1) physician-family health behavior conversations during well-child visits, and (2) four monthly visits with a RDN to evaluate, educate, and implement improved feeding habits and nutritional choices. A third optional component of the intervention included counseling sessions with a social worker to help families overcome barriers to change, such as food security, family relationships, and general parenting strategies (i.e., authoritative vs. authoritarian, permissive, or uninvolved) [22,23]. 

Families were informed of the option to meet with a social worker for a behavioral counseling visit, and those interested were scheduled for a separate appointment with the social worker at the pediatric office. Visits lasted approximately one hour. Behavioral counseling visits were designed to help enable families to incorporate the behavior changes recommended by the RDNs during their sessions. Parents were counseled regarding authoritative parenting practices, such as how to be warm and nurturing while also providing firm and consistent expectations and opportunities for independence. For example, parents were encouraged to establish rules regarding meal times and food choices, while also being sensitive to the child’s satiety cues and supportive when the child tried new foods.

Prior to the intervention, physicians from treatment sites participated in a training seminar from study investigators to improve the content and delivery of their interactions with families regarding healthy lifestyle choices. The training included methods for evaluating and discussing obesity-related lifestyle behaviors with families of preschool-aged children, including healthy nutrition, physical activity, sleep, and sedentary behaviors. Physicians were also instructed regarding appropriate goal-setting strategies, including a brief discussion of motivational interviewing techniques. 

RDNs specializing in pediatric nutrition and trained in motivational interviewing met with intervention parents/caregivers during four educational visits over a six-month period. To maximize treatment consistency, researchers followed an outlined script to provide consistent messages to patients enrolled in the study. Health behavior education focused on the “Healthy Counts” messaging, which includes nine health behaviors (>8 h of sleep, 7 breakfasts/week; 6 home cooked meals/week; 5 servings of fruit and vegetables/day; 4 positive self-messages/day; 3 servings of low fat dairy/day; 2 h or less of screen time/day; 1 h or more of physical activity/day, and 0 sugar-sweetened beverages/day) [24]. Other covered topics included division of responsibility with feeding [25,26], self-regulation, MyPlate [27], meal planning, healthy snacks, daily nutrient needs, picky eating, goal setting and any other specific nutritional concerns. RDNs provided small incentives such as stickers, MyPlate child plates, beach balls, books, and fruit and vegetable plush characters to further support behavior change at the end of each visit. A team of registered dietitians, pediatricians, and childhood-obesity researchers created educational handouts tailored to each well-child visit from 2–5 years of age and each of the four RDN visits, which were distributed by physicians during well-child visits and RDNs during the appropriate appointments.

### 2.4. Measures

Demographics—Basic demographic characteristics were assessed via questionnaire at baseline, and included age, sex, and race/ethnicity of the child, and education level for the mother and father (or respective caregiver). 

Anthropometry—Patient age, height, and weight were obtained at baseline and follow up by trained medical staff at the primary care office after the removal of shoes and excess patient clothing. BMI was calculated as kg/m^2^, and age- and sex-specific BMI was calculated as BMI z-scores and percent of the 95th percentile (%BMI_p95_) using standardized CDC growth curves [28]. %BMI_p95_ expresses BMI as a percentage of the 95th percentile for a child’s age and sex, such that a child with a BMI at the 95th percentile would have a %BMI_p95_ =100%. Weight status was categorized as normal weight (%BMI <85th), overweight (%BMI: 85th–<95th), obesity (%BMI_p95_: 100%–<120%), and severe obesity (%BMI_p95_: ≥120%). 

Child/Family Behaviors—All behavioral assessments were completed at baseline and follow up in order to allow for the evaluation of behavior changes during the intervention. A parent-reported lifestyle questionnaire was used to assess the following child behaviors: moderate- to vigorous-intensity physical activity (MVPA), screen time (including watching television, playing video games, and using cell phones, tablets, and computers), and sleep duration (estimated from typical sleep/wake times). The intensity of MVPA was defined to include activities “where he/she is sweating and breathing hard”. Estimates for MVPA, screen time, and sleep were assessed separately for weekdays and weekends.

Family Nutrition and Physical Activity (FNPA)—Parents also completed the Family Nutrition and Physical Activity (FNPA) screening tool, a validated survey used to assess obesity-related risk factors in the home environment [29]. The FNPA includes twenty questions covering ten subscales that assess a wide range of child health behaviors, including family meal patterns (eating together and eating breakfast), family eating habits (TV during meals and fast food consumption), food choices (ready-to-eat meals and fruit and vegetable intake), beverage choices (sugary drinks and low-fat milk intake), restriction/reward (monitoring “junk” food and using food as a reward), screen time (TV/video game quantity and limit setting), healthy environment (TV in the bedroom and physical activity opportunities), family physical activity (physical activity encouragement and family participation), child physical activity (physical activity during free time and sports participation), and sleep routine (bedtime routine and sleep quantity). Each of the twenty questions includes four response options (1–4 points), ranging from “almost never” to “almost always”. Six questions are reverse coded in order to reduce social desirability bias, but all items are scored such that higher points represented healthier scores. Thus, total scores for the 20 items range from 20 to 80. Subscales are made up of two questions each, and question scores are combined such that total subscale scores range from 2–8 points. The FNPA has demonstrated predictive validity [30], has differentiated between weight status groups from normal weight through severe obesity [31,32], and seems particularly well-suited to predicting increased adiposity risk in young children [33].

Feeding Practices—The Feeding Practices and Structure Questionnaire (FPSQ) was used to assess changes in feeding practices and meal environment structure in participant families. The FPSQ is a 40-item survey that has demonstrated acceptable validity and internal reliability in parents of young children [34]. FPSQ questions load onto nine feeding constructs, including five non-responsive feeding practices scales and 4 structure-related feeding scales. Response options range from 1–5 based on two Likert scales: “never” to “always” and “disagree” to “agree”. Feeding constructs are scored as an average of the individual questions within the subscale, and therefore, also range from 1–5. Non-responsive feeding practices include distrust in appetite (e.g., regulating how much the child eats), overt restriction (e.g., keeping junk foods out of the child’s reach), persuasive feeding (e.g., insisting or showing disapproval when the child isn’t eating), reward for eating (e.g., offering a food/nonfood reward for eating), and reward for behavior (e.g., offering food as a reward for good behavior or to soothe). Structure-related feeding scales include family meal setting (e.g., the child eats meals with the family), structured meal setting (e.g., the child eats meals at the table), structured meal timing (e.g., deciding the times when the child eats), and covert restriction (e.g., avoiding bringing “junk” foods into the house). A reduced 28-item version of the FPSQ (FPSQ-28), using eight of nine original feeding constructs (and removing distrust in appetite) has also been validated and shown to be stable and over time in 2–5 year-olds [35], and provides comparable outcomes when reported by fathers or mothers [36]. The primary FPSQ outcome analysis in the current study included the original 40-item, nine-construct FPSQ, as originally planned, but a secondary analysis comparing outcomes using the reduced 28-item version of the FPSQ was also performed.

### 2.5. Data Analyses

Descriptive characteristics were expressed as means, standard deviations, and frequencies. Differences in baseline characteristics between treatment groups were assessed using independent t-tests for continuous variables and chi-square for categorical measures. Within-group changes in patient characteristics from pre-intervention to post-intervention were evaluated using dependent t-tests. Differences between treatment and control group changes were assessed using two-way repeated measures analysis of variance (RM-ANOVA). Outcome analyses were assessed using two protocols. An as-treated approach was implemented to assess the impact of the treatment among participants who completed both pre-intervention and post-intervention assessments, regardless of the extent of treatment participation. An intention-to-treat (ITT) approach was implemented using a baseline carried forward analysis in which missing post-intervention values were imputed with pre-intervention values in order to minimize potential selection bias due to participant attrition. Alpha was set at 0.05, and SAS software (version 9.4, SAS Institute, Cary, NC, USA) was used for all statistical analyses.

## 3. Results

Participants included 165 parent/child dyads enrolled across treatment (*n* = 93) and control sites (*n* = 72). Youth ranged in age from 2.0–5.9 years (mean (±SD): 3.6 ± 1.0 years) at baseline, and were primarily white (90%), just over half female (56%), and equally classified as having overweight (48%) or obesity (48%), with few having normal weight with rapid BMI increases (5%). Most parents were well educated, with 60% of mothers and 58% of fathers obtaining at least a college degree. All measured demographic characteristics were similar for both youth and parents at baseline across treatment and control groups (Table 1). 

A total of 127 dyads completed follow up measures (77% retention) after an average study period of 6.6 ± 1.9 months, which was similar between treatment (6.4 ± 2.3 months) and control (6.9 ± 1.3 months) families. Retention rates did not significantly differ between treatment (72%) and control (83%) families, or between patients with normal weight/overweight (74%) vs. obesity (79%). Treatment families attended an average of 2.5 ± 1.7 RD visits, and 53% completed all 4 visits. A total of 17 treatment families (18%) chose to attend behavioral counseling sessions with a social worker.

At baseline, patient behavioral measures were similar between treatment and control groups for sleep, physical activity, and screen time, but not for FNPA, which was higher/healthier among controls (*p* = 0.002). During the study period, control patients did not have a significant change in any behaviors. Among treatment patients, FNPA increased 4.6 points (95% confidence interval [CI]: 3.8, 5.8) (*p* < 0.001) and screen time decreased 0.9 h/day (CI: −1.3, −0.4) (*p* < 0.001). When comparing behavior changes between groups, treatment participants improved FNPA (*p* < 0.001) and reduced screen time (*p* < 0.001) significantly more than control patients, while changes in sleep and physical activity did not differ (Table 2). 

Patient BMI, %BMI_p95_, and BMI z-score did not differ between treatment and control patients at baseline. During the program, BMI decreased by −0.2 kg/m^2^ (CI: −0.4, −0.0) (*p* = 0.028) among participants as a whole, but BMI changes did not differ between groups (Table 2). Overall, BMI z-score decreased, on average, among all patients (mean: −0.09 (CI: −0.17, −0.02)) and among treatment patients (mean: −0.13 (CI: −0.23, −0.02), but not among control patients (mean: −0.05 (CI: −0.16, 0.06)). Differences in BMI z-score changes between treatment and control groups were not significant (*p* = 0.332). %BMI_p95_ did not significantly change during the intervention (mean: −0.6 (CI: −1.5, 0.4), and %BMI_p95_ changes did not differ between treatment (mean: −0.8 (CI: −2.1, 0.5)) and control groups (mean: −0.3 (CI: −1.6, 1.0)). 

According to FPSQ scores, baseline feeding practices were similar between treatment and control parents with the exception of overt restriction (*p* < 0.001), which was lower/healthier among controls, and structured meal timing (*p* = 0.012), which was higher/healthier among controls. When comparing feeding practice changes, treatment parents reported greater decreases in non-responsive feeding practices compared to controls, including a larger reduction in distrust in appetite (*p* = 0.015) and reward for behavior (*p* = 0.006) (Table 3). One structure-related feeding practice, structured meal timing, also improved more among treatment vs. control families (*p* < 0.001).

Outcomes from the reduced FPSQ-28 matched those of the original FPSQ. Distrust in appetite was no longer included as a feeding construct in the FPSQ-28, but reward for behavior (*p* = 0.026) and structured meal timing (*p* < 0.001) remained significant. 

Intention-to-treat results, using a “baseline carried forward” analysis, were similar to as-treated results for anthropometric measures, health behaviors, and parent feeding practices. In particular, all statistically significant and non-significant results using an as-treated approach remained unchanged using an ITT approach, though the effect sizes of the changes were slightly reduced. Based on ITT results, screen time decreased 0.6 h/day (CI: −0.9, −0.3) among treatment patients vs. an increase of 0.2 h/day (CI: 0.0, 0.4) among controls (*p* < 0.001), and FNPA increased 2.6 points (CI: 1.7, 3.5) in treatment patients vs. 0.1 point (CI: −0.7, 0.8) in controls (*p* < 0.001). For feeding practices, treatment parents reported greater decreases in distrust in appetite (*p* = 0.024) and reward for behavior (*p* = 0.013), and greater increases in structured meal timing (*p* = 0.015) using ITT, similar to as-treated results. 

## 4. Discussion

In the current study, families who completed the 6-month intervention demonstrated meaningful improvements in child health behaviors and parent feeding practices when compared to control families. For children who completed treatment, these changes included a 4.6-point improvement in FNPA scores and a nearly one-hour reduction in screen time, on average. We have previously shown that, among children and adolescents with overweight/obesity, each FNPA-point difference is associated with a 0.12 kg/m^2^ difference in BMI, after adjusting for age and sex [32]. Furthermore, youth with class III severe obesity (%BMI_p95_ ≥ 140) have twice the odds of low (at-risk) FNPA scores when compared to children with overweight/obesity. Thus, a mean increase of over 4 points in FNPA scores represents a meaningful improvement in health behaviors, and if maintained, may contribute to future improvements in adiposity. Screen time has also been linked to excess adiposity in this age group, both cross-sectionally [37,38,39,40,41,42,43,44] and prospectively [45,46], suggesting screen time habits of preschoolers are predictive of current BMI and future adiposity changes. In the current study, parents of both treatment and control families reported over two hours of screen time per day in their preschool child, which is over double the current recommendation for this age group [47]. Thus, the mean 0.9 h/day decrease in screen time among treatment children represents an almost 40% reduction in duration and an important positive lifestyle change. 

Perhaps most notably, parents who participated in the study intervention improved specific feeding practices in comparison to control parents, including lower appetite distrust, less rewarding with food, and greater consistency in meal timing. These findings are important in light of the available literature linking parent feeding practices to children’s eating behaviors and adiposity. Cross-sectional studies have established positive associations between BMI and food restriction [48,49] and rewarding with food (i.e., instrumental feeding) [50], and inverse associations between BMI and pressure to eat [48,49,51]. However, interpretation of these findings is difficult due to the interactive and reciprocal nature of parental feeding, child eating, and child adiposity. For example, children perceived to eat and weigh less than peers may be more likely to be pressured to eat, whereas perceptions of overeating and overweight in children may lead to increased parental restriction. This hypothesis has been confirmed by longitudinal research, which has demonstrated associations between lower weights and increases in pressure to eat as well as between higher weights and decreases in pressure to eat and increases in food restriction [52,53,54].

Steinsbekk et al. prospectively evaluated parent feeding practices and children’s eating behavior from ages 6–8 in a cohort of Norwegian children, and found that instrumental feeding at age 6 predicted elevated food responsiveness (i.e., tendency to want to overeat in response to highly palatable food) and emotional overeating two years later [55]. In addition, parental encouragement to eat predicted increases in children’s enjoyment of food. Rodgers et al. in a study of preschool children, found that instrumental feeding practices were prospectively associated with increases in BMI *z*-scores one year later, and that parental restriction, emotional feeding, and encouragement to eat predicted children’s emotional eating, tendency to overeat, and food approach behaviors [13]. A recent prospective study, incorporating the same FPSQ assessment tool as the current study, found that lower covert restriction and higher reward for behavior when children were two years old was prospectively associated with higher food responsiveness in children approximately 1.5 years later [15]. These findings suggest that parent feeding practices have the potential to influence children’s future eating behaviors, attitudes towards foods, and BMI. Thus, treatment approaches, such as those of the current study, which teach parents to avoid external prompts to eat (i.e., non-responsive feeding practices), and instead allow children to rely on internal hunger and satiety signals appear to be an appropriate target for promoting healthier eating behaviors and weights among young children. 

Yet, few studies have investigated the feasibility of influencing parenting feeding behaviors among preschool-aged youth, and even fewer have done so in a primary care setting. The NOURISH randomized controlled trial used a series of group sessions delivered at child health clinics in Australia, and demonstrated improvement in responsive feeding practices in mothers of children through two years of age, including less pressured and emotional feeding and less rewarding with food [16]. Follow up outcomes of NOURISH found that these improved feeding practices were maintained through five years of age, though no differences in weight or BMI outcomes were apparent at any age studied [17]. In a randomized controlled trial of the Parents and Tots Together intervention, researchers administered nine educational group sessions to parents of 2–5 year olds, resulting in decreased use of restrictive feeding behaviors in treatment parents, but no change in pressured feeding or child BMI [18]. Similarly, the Kids and Adults Now—Defeat Obesity! (KAN-DO) trial showed reduced instrumental feeding and emotional feeding in mothers of 2–5 year olds after receiving eight monthly interactive kits and telephone coaching sessions, though child weight was unaffected [19]. Treatment parents in the current study also reduced instrumental feeding through a reduction in rewarding with food, and moved towards increased use of appetite for feeding. Taken together, these findings suggest that a low-intensity, primary care intervention can be effective in improving parental feeding behaviors. 

Despite these behavioral improvements, no significant differences in BMI, BMI z-score or %BMI_p95_ changes were evident between treatment and control groups. The significant decrease in BMI among treatment and control patients, as a whole, may have been influenced by the age group of the sample, many of whom may have not reached their lowest BMI (i.e., adiposity rebound), which occurs, on average, at 5 years of age among U.S. children [56]. However, elevated BMI is associated with an earlier adiposity rebound [8], suggesting the age of BMI nadir in the current sample of children with overweight/obesity is likely younger than typical in US children. In our sample, 62% of youth ≥5 years of age increased their BMI during the study period, whereas only 27% of children <4 years experienced BMI gains. Therefore, changes in age- and sex-adjusted BMI z-scores and %BMI_p95_ are more appropriate metrics for comparison across participants.

The small decrease in BMI z-score among treatment participants in our study is similar to findings from a recent review of controlled, primary care pediatric weight management studies, which found that, among the 18 studies reviewed, 17 showed a positive effect, such that treatment improved more than control, but only four of these treatment effects were statistically significant [57]. When combined in a meta-analysis, the net effect was a small, significant, positive effect size (Cohen’s d = 0.26 (CI: 0.14, 0.38)). In addition, the number of treatment contacts and pediatrician visits were significant moderators, with more visits resulting in greater treatment effects. These studies included 11.6 treatment contacts, on average, whereas the current study included only four dietitian visits plus typical physician well-child visits over a six-month period. Thus, it is possible that increasing the number of treatment contacts in the current study may have resulted in larger adiposity reductions among treatment patients. 

Another recent meta-analysis by Sim et al. found similar results when evaluating primary care pediatric weight management programs delivered onsite by primary care staff members [58]. When combined, the 12 studies reviewed were associated with a small, but significant decrease in BMI z-scores (−0.04 (CI: −0.08, −0.01)). The current study showed a similar, yet non-significant difference in BMI z-score changes between treatment and control groups (mean: −0.07, CI: −0.23, 0.08). When considered independently, these changes in zBMI are marginal, and would likely have little impact on long-term health. However, current guidelines recommend stage-based treatment for childhood obesity, which includes escalating levels of treatment intensity across stages [59]. Thus, low-intensity, behavior-based programs in the primary care setting (i.e., stage 1), may provide utility as a first step within a broader, comprehensive effort to impact childhood obesity in the health care setting. 

This study has several limitations. We used a non-randomized study design by grouping treatment and control patients based on their primary care provider in order to simplify treatment administration and to prevent contamination bias. This quasi-experimental design introduces a potential selection bias wherein treatment patients may differ from control patients; however, several steps were taken in order to minimize this potential bias. First, the primary care sites were selected for study inclusion based on similar patient demographics. Second, patient characteristics were compared at baseline and found to be similar across groups. Characteristics were not significantly different between groups for any demographic or behavioral variables, with the exception of FNPA. Lastly, repeated measures ANOVA, which takes into account potential baseline differences, was used to compare changes in treatment and control group outcomes. Yet, it is possible that unmeasured factors could have had a differential influence on study groups. In addition, despite relatively high retention rates (77%) and similar rates between treatment and control groups, patient attrition represents a potential bias. To minimize this attrition bias, we included all available data in our as-treated analyses, regardless of treatment participation, and included an intention-to-treat analysis which substituted baseline values for missing outcomes at follow up. Even so, study outcomes can only be generalized to patients who attend primary care well-child visits, and who are interested in participating in a health-behavior treatment program. Another limitation includes the self-reported nature of both parent feeding practices and child behaviors, which although collected using validated tools, cannot completely eliminate potential social-desirability bias. To minimize this bias, outcomes were collected using the same methodology across all sites, including anthropometry and survey administration in primary care offices by staff members who were not involved in the program. Lastly, our study included families who, due to geographical and clinic demographics, were mostly white and well-educated, which limits the generalizability of our findings beyond this population. 

There is urgent need for effective pediatric primary care programs that can spearhead obesity prevention and treatment efforts for children of all ages. Our study demonstrates meaningful improvements in obesogenic risk factors in the home environment, healthier parental feeding practices, and reduced child screen time after a 6-month, low-intensity primary care health behavior intervention. Despite non-significant differences in short-term child BMI changes, future improvements remain feasible among treatment families if improvements in health habits and BMI trajectories are maintained. Such longer-term outcome assessments are currently underway. Additional research is also needed to prospectively evaluate the reciprocal effects of parent feeding practices and child eating behaviors, including how these habits can be influenced through treatment and prevention programs, the durability of these behavior changes, and their impact on children’s growth patterns. 

## Figures and Tables

**Table 1:** Baseline patient characteristics among children 2–5 years participating in a primary care weight management program.

	Total		Treatment		Control		
	(*n* = 165)		(*n* = 93)		(*n* = 72)		
	Mean	(SD)	Mean	(SD)	Mean	(SD)	*p*-Value
Age (year), Mean (SD)	3.6	(1.0)	3.7	(1.0)	3.5	(1.0)	0.754
Height (cm), Mean (SD)	101.3	(8.9)	102.2	(8.9)	100.1	(8.9)	0.963
Weight (kg), Mean (SD)	19.3	(4.3)	19.6	(4.4)	18.9	(4.1)	0.632
BMI (kg/m^2^), Mean (SD)	18.6	(1.7)	18.6	(1.6)	18.7	(1.9)	0.069
%BMI_p95_, ^1^ Mean (SD)	102.0	(9.4)	101.9	(8.7)	102.1	(10.4)	0.109
Child sex, %	**%**		**%**		**%**		0.632
Boys	44.3		42.6		46.1		
Girls	55.7		57.5		53.9		
Child race, %	**%**		**%**		**%**		0.346
Black	2.3		2.3		2.3		
White	90.3		88.5		92.1		
Hispanic	3.4		5.8		1.1		
Other	4.0		3.5		4.5		
Mother’s Education, %	**%**		**%**		**%**		0.280
Some college or less	40.0		43.8		36.0		
College grad or more	60.0		56.3		64.0		
Father’s education, %	%		%		%		0.650
Some college or less	42.2		43.8		40.5		
College grad or more	57.8		56.3		59.6		
Weight Status, %	%		%		%		0.520
Normal Weight ^2^	5.0		5.4		4.4		
Overweight ^3^	47.5		43.5		52.9		
Obesity ^4^	42.5		45.7		38.2		
Severe Obesity ^5^	5.0		5.4		4.4		

^1^ Percent of the 95th BMI percentile. ^2^ BMI percentile <85th), ^3^ BMI percentile 85th–<95th), ^4^ Percent of the 95th BMI percentile 100%–<120%, ^5^ Percent of the 95th BMI Percentile ≥120%).

**Table 2:** Changes in body mass index (BMI) and behaviors among children 2–5 years participating in a primary care weight management program.

	Pre-Intervention		Post-Intervention		Change		
	Mean	(SD)	Mean	(SD)	Mean	(SD)	*p*-Value
**BMI (kg/m^2^)**							0.879
Control (*n* = 54)	18.6	(1.7)	18.4	(1.9)	−0.2	(0.9)	
Treatment (*n* = 65)	18.6	(1.6)	18.4	(2.0)	−0.2	(1.0)	
**%BMI_p95_, ^1^ %**							0.619
Control (*n* = 53)	101.4	(9.0)	101.1	(10.1)	−0.3	(4.7)	
Treatment (*n* = 66)	102.0	(8.5)	101.2	(10.1)	−0.8	(5.3)	
**BMI *z*-score**							0.332
Control (*n* = 53)	1.66	(0.63)	1.60	(0.70)	−0.05	(0.40)	
Treatment (*n* = 66)	1.71	(0.55)	1.59	(0.68)	−0.13 *	(0.42)	
**Sleep (h/day)**							0.929
Control (*n* = 59)	10.7	(0.7)	10.8	(0.7)	0.1	(0.8)	
Treatment (*n* = 47)	10.7	(0.8)	10.8	(0.9)	0.1	(0.6)	
**Physical Activity (min/day)**							0.377
Control (*n* = 58)	109.9	(69.5)	109.9	(70.6)	−0.1	(73.6)	
Treatment (*n* = 47)	98.3	(73.0)	109.7	(75.2)	11.4	(55.1)	
**Screen Time (h/day)**							<0.001 ^†^
Control (*n* = 54)	2.1	(1.1)	2.4	(1.1)	0.3	(1.1)	
Treatment (*n* = 58)	2.4	(1.7)	1.5	(1.3)	−0.9 *	(1.8)	
**FNPA (total score)**							<0.001 ^†^
Control (*n* = 64)	67.2	(6.6)	67.3	(6.1)	0.1	(4.2)	
Treatment (*n* = 48)	64.9	(6.3)	69.5	(5.5)	4.6 *	(4.6)	

^1^ Percent of the 95th BMI percentile.* Significant within-group change pre-intervention to post-intervention according to dependent *t*-test (*p* < 0.05). ^†^ Significantly different change between groups according to RM-ANOVA (*p* < 0.05).

**Table 3:** Changes in feeding practices according to the Feeding Practices and Structure Questionnaire subscales among parents of children 2–5 years participating in a primary care pediatric weight management program.

	Pre-Intervention		Post-Intervention		Change		
	Mean	(SD)	Mean	(SD)	Mean	(SD)	*p*-Value
**Distrust in Appetite**							0.015 ^†^
Control	2.9	(0.6)	2.9	(0.6)	0.0	(0.6)	
Treatment	2.6	(0.5)	2.4	(0.6)	−0.2 *	(0.6)	
**Reward for Behavior**							0.006 ^†^
Control	2.2	(0.6)	2.2	(0.7)	0.0	(0.5)	
Treatment	1.9	(0.7)	1.7	(0.5)	−0.3 *	(0.5)	
**Reward for Eating**							0.053
Control	2.3	(0.7)	2.2	(0.6)	−0.1 *	(0.5)	
Treatment	2.2	(0.7)	1.9	(0.7)	−0.3 *	(0.5)	
**Persuasive Feeding**							0.295
Control	3.1	(0.6)	3.0	(0.6)	−0.1	(0.5)	
Treatment	2.9	(0.6)	2.7	(0.7)	−0.2 *	(0.5)	
**Overt Restriction**							0.524
Control	3.7	(0.8)	3.5	(1.0)	−0.1	(0.9)	
Treatment	4.1	(0.8)	3.9	(0.8)	−0.2	(0.9)	
**Covert Restriction**							0.883
Control	3.4	(0.8)	3.4	(0.7)	0.1	(0.6)	
Treatment	3.5	(0.7)	3.6	(0.6)	0.1	(0.8)	
**Structured Meal Setting**							0.088
Control	4.4	(0.7)	4.5	(0.6)	0.1	(0.5)	
Treatment	4.4	(0.6)	4.6	(0.4)	0.2 *	(0.5)	
**Structured Meal Timing**							<0.001 ^†^
Control	4.0	(0.6)	3.9	(0.7)	−0.1	(0.6)	
Treatment	3.7	(0.6)	4.1	(0.6)	0.4 *	(0.6)	
**Family Meal Setting**							0.692
Control	4.4	(0.7)	4.5	(0.6)	0.1	(0.5)	
Treatment	4.3	(0.7)	4.4	(0.6)	0.1	(0.5)	

* Significant within-group change pre-intervention to post-intervention according to dependent *t*-test (*p* < 0.05). ^†^ Significantly different change between groups according to RM-ANOVA (*p* < 0.05).

## References

[1] Ogden C.L., Carroll M.D., Flegal K.M. (2008). High body mass index for age among US children and adolescents, 2003–2006. JAMA.

[2] Katzmarzyk P.T., Srinivasan S.R., Chen W., Malina R.M., Bouchard C., Berenson G.S. (2004). Body mass index, waist circumference, and clustering of cardiovascular disease risk factors in a biracial sample of children and adolescents. Pediatrics.

[3] Smoak C.G., Burke G.L., Webber L.S., Harsha D.W., Srinivasan S.R., Berenson G.S. (1987). Relation of obesity to clustering of cardiovascular disease risk factors in children and young adults—The Bogalusa Heart Study. Am. J. Epidemiol..

[4] Power C., Lake J.K., Cole T.J. (1997). Measurement and long-term health risks of child and adolescent fatness. Int. J. Obes..

[5] Kaplan E.L., Rocchini A.R. (1991). Cardiovascular disease in children: Reflections on the last half century and expectations for the future. Minn. Med..

[6] Serdula M.K., Ivery D., Coates R.J., Freedman D.S., Williamson D.F., Byers T. (1993). Do obese children become obese adults? A review of the literature. Prev Med..

[7] Cunningham S.A., Kramer M.R., Narayan K.M. (2014). Incidence of childhood obesity in the United States. N. Engl. J. Med..

[8] Cole T.J. (2004). Children grow and horses race: Is the adiposity rebound a critical period for later obesity?. BMC Pediatr..

[9] Claris O., Beltrand J., Levy-Marchal C. (2010). Consequences of intrauterine growth and early neonatal catch-up growth. Semin. Perinatol..

[10] Dulloo A.G., Jacquet J., Seydoux J., Montani J.P. (2006). The thrifty ‘catch-up fat’ phenotype: Its impact on insulin sensitivity during growth trajectories to obesity and metabolic syndrome. Int. J. Obes..

[11] Ong K.K., Ahmed M.L., Emmett P.M., Preece M.A., Dunger D.B. (2000). Association between postnatal catch-up growth and obesity in childhood: Prospective cohort study. BMJ.

[12] Taylor R.W., Grant A.M., Goulding A., Williams S.M. (2005). Early adiposity rebound: Review of papers linking this to subsequent obesity in children and adults. Curr. Opin. Clin. Nutr. Metab. Care.

[13] Rodgers R.F., Paxton S.J., Massey R., Campbell K.J., Wertheim E.H., Skouteris H., Gibbons K. (2013). Maternal feeding practices predict weight gain and obesogenic eating behaviors in young children: A prospective study. Int. J. Behav. Nutr. Phys. Act..

[14] Steinsbekk S., Llewellyn C.H., Fildes A., Wichstrøm L. (2017). Body composition impacts appetite regulation in middle childhood. A prospective study of Norwegian community children. Int. J. Behav. Nutr. Phys. Act..

[15] Jansen E., Williams K.E., Mallan K.M., Nicholson J.M., Daniels L.A. (2018). Bidirectional associations between mothers’ feeding practices and child eating behaviours. Int. J. Behav. Nutr. Phys. Act..

[16] Daniels L.A., Mallan K.M., Battistutta D., Nicholson J.M., Perry R., Magarey A. (2012). Evaluation of an intervention to promote protective infant feeding practices to prevent childhood obesity: Outcomes of the NOURISH RCT at 14 months of age and 6 months post the first of two intervention modules. Int. J. Obes..

[17] Daniels L.A., Mallan K.M., Nicholson J.M., Thorpe K., Nambiar S., Mauch C.E., Magarey A. (2015). An Early Feeding Practices Intervention for Obesity Prevention. Pediatrics.

[18] Haines J., Rifas-Shiman S.L., Gross D., McDonald J., Kleinman K., Gillman M.W. (2016). Randomized trial of a prevention intervention that embeds weight-related messages within a general parenting program. Obesity.

[19] Østbye T., Krause K.M., Stroo M., Lovelady C.A., Evenson K.R., Peterson B.L., Bastian L.A., Swamy G.K., West D.G., Brouwer R.J. (2012). Parent-focused change to prevent obesity in preschoolers: Results from the KAN-DO study. Prev. Med..

[20] Ekelund U., Ong K., Linné Y., Neovius M., Brage S., Dunger D.B., Wareham N.J., Rössner S. (2006). Upward weight percentile crossing in infancy and early childhood independently predicts fat mass in young adults: The Stockholm Weight Development Study (SWEDES). Am. J. Clin. Nutr..

[21] Hagan J.F., Shaw J.S., Duncan P.M. (2017). Bright Futures: Guidelines for Health Supervision of Infants, Children and Adolescents.

[22] Baumrind D. (1966). Effects of authoritative parental control on child behavior. Child Dev..

[23] Maccoby E.E., Martin J.A., Mussen P.H. (1983). Socialization in the Context of the Family: Parent-Child Interaction. Handbook of Child Psychology.

[24] Tucker J.M., Eisenmann J.C., Howard K., Guseman E.H., Yee K.E., DeLaFuente K., Graybill J., Roberts M., Murphy M., Saturley H. (2014). FitKids360: Design, conduct, and outcomes of a stage 2 pediatric obesity program. J. Obes..

[25] Satter E., O’Donahue W., Moore B.A., Scott B. (2007). The Satter Feeding Dynamics Model of child overweight definition, prevention and intervention. Pediatric and Adolescent Obesity Treatment: A Comprehensive Handbook.

[26] Satter E.M. (1986). The feeding relationship. J. Am. Diet. Assoc..

[27] ChooseMyPlate United States Department of Agriculture Human Nutrition Information Service. https://www.choosemyplate.gov/.

[28] Kuczmarski R.J., Ogden C.L., Guo S.S., Grummer-Strawn L.M., Flegal K.M., Mei Z., Wei R., Curtin L.R., Roche A.F., Johnson C.L. (2002). 2000 CDC Growth Charts for the United States: Methods and development. Vital Health Stat. 11.

[29] Ihmels M.A., Welk G.J., Eisenmann J.C., Nusser S.M. (2009). Development and preliminary validation of a Family Nutrition and Physical Activity (FNPA) screening tool. Int. J. Behav. Nutr. Phys. Act..

[30] Ihmels M.A., Welk G.J., Eisenmann J.C., Nusser S.M., Myers E.F. (2009). Prediction of BMI change in young children with the FamilyNutrition and Physical Activity (FNPA) screening tool. Ann. Behav. Med..

[31] Yee K.E., Pfeiffer K.A., Turek K., Bakhoya M., Carlson J.J., Sharman M., Lamb E., Eisenmann J.C. (2015). Association of the Family Nutrition and Physical Activity Screening Tool with Weight Status, Percent Body Fat, and Acanthosis Nigricans in Children from a Low Socioeconomic, Urban Community. Ethn. Dis..

[32] Tucker J.M., Howard K., Guseman E.H., Yee K.E., Saturley H., Eisenmann J.C. (2017). Association between the Family Nutrition and Physical Activity Screening Tool and obesity severity in youth referred to weight management. Obes. Res. Clin. Pract..

[33] Peyer K.L., Welk G.J. (2017). Construct Validity of an Obesity Risk Screening Tool in Two Age Groups. Int. J. Environ. Res. Public Health.

[34] Jansen E., Mallan K.M., Nicholson J.M., Daniels L.A. (2014). The feeding practices and structure questionnaire: Construction and initial validation in a sample of Australian first-time mothers and their 2-year olds. Int. J. Behav. Nutr. Phys. Act..

[35] Jansen E., Williams K.E., Mallan K.M., Nicholson J.M., Daniels L.A. (2016). The feeding practices and structure questionnaire (FPSQ-28): A parsimonious version validated for longitudinal use from 2 to 5 years. Appetite.

[36] Jansen E., Harris H.A., Mallan K.M., Daniels L., Thorpe K. (2018). Measurement invariance of the Feeding Practices and Structure Questionnaire-28 among a community of socioeconomically disadvantaged mothers and fathers. Appetite.

[37] Ariza A.J., Chen E.H., Binns H.J., Christoffel K.K. (2004). Risk factors for overweight in five- to six-year-old Hispanic-American children: A pilot study. J. Urban Health.

[38] Cox R., Skouteris H., Rutherford L., Fuller-Tyszkiewicz M., Dell’ Aquila D., Hardy L.L. (2012). Television viewing, television content, food intake, physical activity and body mass index: A cross-sectional study of preschool children aged 2–6 years. Health Promot. J. Austr..

[39] Jackson D.M., Djafarian K., Stewart J., Speakman J.R. (2009). Increased television viewing is associated with elevated body fatness but not with lower total energy expenditure in children. Am. J. Clin. Nutr..

[40] Janz K.F., Levy S.M., Burns T.L., Torner J.C., Willing M.C., Warren J.J. (2002). Fatness, physical activity, and television viewing in children during the adiposity rebound period: The Iowa Bone Development Study. Prev. Med..

[41] Jiang J., Rosenqvist U., Wang H., Greiner T., Ma Y., Toschke A.M. (2006). Risk factors for overweight in 2- to 6-year-old children in Beijing, China. Int. J. Pediatr. Obes..

[42] Manios Y., Kourlaba G., Kondaki K., Grammatikaki E., Anastasiadou A., Roma-Giannikou E. (2009). Obesity and television watching in preschoolers in Greece: The GENESIS study. Obesity.

[43] Mendoza J.A., Zimmerman F.J., Christakis D.A. (2007). Television viewing, computer use, obesity, and adiposity in US preschool children. Int. J. Behav. Nutr. Phys. Act..

[44] Müller M.J., Koertzinger I., Mast M., Langnäse K., Grund A. (1999). Physical activity and diet in 5 to 7 years old children. Public Health Nutr..

[45] Jago R., Baranowski T., Baranowski J.C., Thompson D., Greaves K.A. (2005). BMI from 3-6 y of age is predicted by TV viewing and physical activity, not diet. Int. J. Obes..

[46] Janz K.F., Burns T.L., Levy S.M. (2005). Tracking of activity and sedentary behaviors in childhood: The Iowa Bone Development Study. Am. J. Prev. Med..

[47] AAP Council on Communications and Media (2016). Media and Young Minds. Pediatrics.

[48] Faith M.S., Scanlon K.S., Birch L.L., Francis L.A., Sherry B. (2004). Parent-child feeding strategies and their relationships to child eating and weight status. Obes. Res..

[49] Ventura A.K., Birch L.L. (2008). Does parenting affect children’s eating and weight status?. Int. J. Behav. Nutr. Phys. Act..

[50] Musher-Eizenman D.R., de Lauzon-Guillain B., Holub S.C., Leporc E., Charles M.A. (2009). Child and parent characteristics related to parental feeding practices. A cross-cultural examination in the US and France. Appetite.

[51] Farrow C.V., Blissett J. (2008). Controlling feeding practices: Cause or consequence of early child weight?. Pediatrics.

[52] Jansen P.W., Tharner A., van der Ende J., Wake M., Raat H., Hofman A., Verhulst F.C., van Ijzendoorn M.H., Jaddoe V.W., Tiemeier H. (2014). Feeding practices and child weight: Is the association bidirectional in preschool children?. Am. J. Clin. Nutr..

[53] Tschann J.M., Martinez S.M., Penilla C., Gregorich S.E., Pasch L.A., de Groat C.L., Flores E., Deardorff J., Greenspan L.C., Butte N.F. (2015). Parental feeding practices and child weight status in Mexican American families: A longitudinal analysis. Int. J. Behav. Nutr. Phys. Act..

[54] Afonso L., Lopes C., Severo M., Santos S., Real H., Durao C., Moreira P., Oliveira A. (2016). Bidirectional association between parental child-feeding practices and body mass index at 4 and 7 y of age. Am. J. Clin. Nutr..

[55] Steinsbekk S., Belsky J., Wichstrøm L. (2016). Parental feeding and child eating: An investigation of reciprocal effects. Child Dev..

[56] Boonpleng W., Park C.G., Gallo A.M. (2012). Timing of adiposity rebound: A step toward preventing obesity. Pediatr. Nurs..

[57] Mitchell T.B., Amaro C.M., Steele R.G. (2016). Pediatric Weight Management Interventions in Primary Care Settings: A Meta-Analysis. Health Psychol..

[58] Sim L.A., Lebow J., Wang Z., Koball A., Murad M.H. (2016). Brief Primary Care Obesity Interventions: A Meta-analysis. Pediatrics.

[59] Barlow S.E. (2007). Committee at Expert Committee Recommendations regarding the prevention, assessment, and treatment of child and adolescent overweight and obesity: Summary report. Pediatrics.

